# Towards a Model of Valued Human Cognitive Abilities: An African Perspective Based on a Systematic Review

**DOI:** 10.3389/fpsyg.2020.538072

**Published:** 2020-12-04

**Authors:** Seth Oppong

**Affiliations:** Department of Psychology, University of Botswana, Gaborone, Botswana

**Keywords:** African models, intelligence, cognitive abilities, Africa, valued human cognitive abilities

## Abstract

Studies that investigate cognitive ability in African children and estimate the general cognitive abilities of African adults tend to work with existing models of intelligence. However, African philosophy and empirical studies in cross-cultural psychology have demonstrated that conceptualizations of human cognitive ability vary with location. This paper begins with the assumption that the existing Anglo-American models of cognitive abilities are valuable but limited in their capacity to account for the various conceptualizations of valued cognitive abilities in different human societies. On the basis of this assumption, I employ extant empirical evidence generated through ethnographic studies across Africa to formulate what an African model of valued human cognitive ability ought to be. The output of this formulation has been so christened a model of valued cognitive ability in order to draw attention to the fact that models of cognitive abilities have currency and values in each human society. This value allocation is expected to influence which elements of cognitive ability each human society will promote and develop. In addition, implications for theory, research and praxes are discussed.

## Introduction

Cognitive ability is a valuable human attribute in so far as it has been shown that it has implications for life outcomes (Serpell, 1993; Lo, 2017; Cofnas, 2020). Opoku (2012, p. 538) has defined cognitive abilities as “all activities generated from within the conscious mind which influence our behavior.” He further argued that it is debatable to equate cognitive abilities to intelligence as the former is broadly defined (Opoku, 2012). In practice, cognitive ability has been conceptualized as a general mental ability (intelligence) or as specific abilities and has been measured either as a single construct or as specific intellectual abilities (Gregory, 2007; Butts, 2014; Fernández-Berrocal and Checa, 2016; Pardeller et al., 2017). Thus, in this paper, the two will be used interchangeably in sync with the current common practice.

Various studies have established that cognitive ability is a significant predictor of academic success, physical health and longevity, and job performance (e.g.,: Judge et al., 2004; Roberts et al., 2007; Strenze, 2007; Lo, 2017). It is important to note that there are methodological concerns (Opoku, 2012; Lo, 2017; Oppong, 2017a) and competing predictors such as parental socioeconomic status and academic performance (Strenze, 2007). For instance, using 85 data sets, Strenze (2007) conducted a meta-analytic review of prospective cohort studies examining the predictive capacity of intelligence, parental socioeconomic status and school grades. Strenze (2007) reached a rather modest conclusion that “while intelligence is one of the central determinants of one's socioeconomic success, parental SES [socioeconomic status] and academic performance also play an important role in the process of status attainment” (pp. 415–416). Opoku (2012) has also called the measurement of cognitive abilities into question. He contended that culture, ecological context, language, and score interpretations are some of the methodological flaws in the measurement of cognitive abilities. Similarly, Oppong (2017a, p. 12) drew attention to the fact the “conceptualizing the construct…is always a ‘victim’ of the developer's cognitive biases (which derives from one's culture through socialization).”

The definition, measurement, and research around intelligence is both popular and controversial (Lo, 2017; Oppong, 2017a; Cofnas, 2020). A more controversial aspect of intelligence research has been the persistent attempts (speculatively or empirically) to explain the group differences in terms of genotypic variance (Deary et al., 2010). From a philosophical pursuit of truth (if there is any such thing as that in science), Cofnas (2020) argues that the verdict is still not out there given that neuroscience research seems to be progressively edging closer to establishing conclusive scientific evidence in favor of hereditarianism as opposed to environmentalism.

While it is true that there is an accumulating evidence base in neuroscience pointing to some genetic basis (Deary et al., 2010), it is still questionable to use a measurement tool whose validity has been challenged (Tyson et al., 2011; Opoku, 2012; Oppong, 2017a) as the basis of the neuroscience of intelligence. Perhaps, Bhaskar (1998, 2008) argument that human sciences based on positivism amounts to naïve realism is tenable here. Empirical regularity or correlation between two sets of observed data (the empirical world) does not necessarily reflect the underlying mechanisms (the real world) which generate the actual event (the actual world) which produce the empirical regularities (see Bhaskar, 1998 detailed exposition on critical realism). Beyond the question of naïve realism, there is the teething problem of epistemological violence (Teo, 2008, 2010; Oppong, 2019). Interpreting the group differences in cognitive abilities the way they are currently may result in structural or indirect violence.

There is a growing charge around the world to begin to make psychology a truly global science (see Jovanović, 2005; Staeuble, 2005; Oppong, 2019), imagining new ways of thinking and researching. Oppong (2019) argues that psychology stands to benefit if alternative views of human nature are developed and encouraged to augment the current narrow perspectives. Brzezinski (2014) and Grzelak (2014) expressed similar view about what psychological science stands to gain if psychology becomes more inclusive assimilating other perspectives including those generated within Polish psychology. Jovanović (2005) and Staeuble (2005) have both argued that the current approach to internationalization of psychology has been in only one direction. Jovanović (2005, p. 78) captures this one-way traffic in these words: “to promote, distribute or impose psychological knowledge of a very specific Western territorial and cultural origin to the other parts of the world territory and socio-cultural landscape.” Jovanović (2005, p. 78) choice of words such as “a very specific Western territorial and cultural origin” indicates that even within the so-called Western world, it appears that a very narrow view of human nature within the varied Western views (the US perspective) is being promoted. Thus, it is important not to even present Western psychology as a homogeneous entity as it is more an American perspective than a Western one (Pickren, 2009; Sam, 2014). Staeuble (2005) has also argued for a global psychological science that is inclusive allowing other voices to be heard. It is against this background that the quest of this special issue to ask of African psychologists “what Africa can do for psychological science” is a relevant question at this point. We are at an epoch where debates are ongoing about the place and role of indigenous psychologies in a global psychology (Nwoye, 2015; Jahoda, 2016; Allwood, 2018; Long, 2019).

In a review of the extant cross-cultural psychology literature on intelligence, Matsumoto and Juang (2007) documented evidence that there are cultural differences in the meaning and the concept of intelligence. Further, Matsumoto and Juang (2007, p. 128) asserted that:

Perhaps the field [of intelligence research] is coming to realize that intelligence in its broadest sense may be more aptly defined as “the skills and abilities necessary to effectively accomplish cultural goals.”

Writing in the *Proceedings of the National Academy of Sciences of the United States of America (PNAS)*, Bradya et al. (2018) argued that the interpretive power of psychology can be expanded by attending to culture. Bradya et al. (2018) specifically outlined the following as approaches to achieving it: culture-conscious research questions, culture-conscious research design, and culture-conscious data analysis and interpretation. In the same issue of *PNAS*, Rada et al. (2018) also outlined some strategies that can make psychology a truly global science of all *Homo sapiens*. The strategies were more at the editorial stage of the publication process, nonetheless they serve as guidelines for reporting with the potential to influence the conduct of research in the future. The purpose of this paper, therefore, is to employ the extant empirical evidence generated through ethnographic and similar field studies across Africa to formulate what an African model of valued human cognitive ability ought to be.

## Western Models of Intelligence and Their Limitations

**Figure 1:**
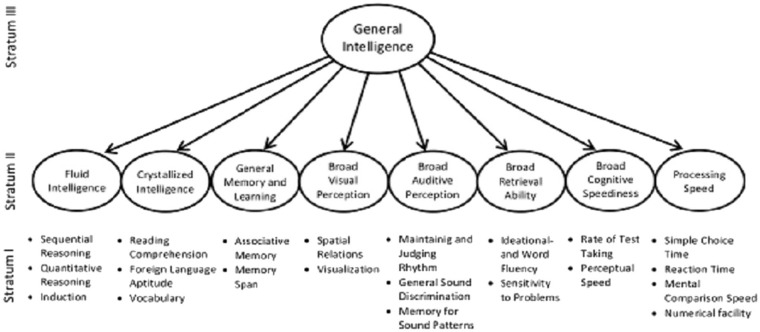
A modified version of Carroll's three-stratum theory. Source: Carroll (1993, p. 626) slightly modified by Wilhelm and Schroeders (2019, p. 259).

Over the years, different models of intelligence have been developed beginning from the days of Alfred Binet in 1905. Thus, the field of intelligence research has evolved dramatically (Carroll, 1993; Sternberg and Kaufman, 1998; Wilhelm and Schroeders, 2019). By far, the most significant milestone in the domain of intelligence research is arguably John B. Carroll's (1993) seminal work (Gregory, 2007; Mcgrew, 2009; Keith and Reynolds, 2010; Wilhelm and Schroeders, 2019). Carroll's (1993) survey of factor-analytic studies on intelligence was based on studies which collected data from the following countries: US, England, Canada, Germany, Australia, Sweden, South Africa, Japan, Netherlands, Norway, Switzerland, Scotland, Argentina, Italy, Ireland, Saudi Arabia, Spain, former Soviet Union (Union of Soviet Socialist Republics), and Yugoslavia (which comprised Bosnia and Herzegovina, Croatia, Macedonia, Montenegro, Serbia, and Slovenia). On the basis of these datasets published between 1925 and 1987, Carroll (1993) proposed his three-stratum theory of cognitive abilities. The three-stratum theory presents general intelligence and its constitution as higher-order factors (Carroll, 1993; Gregory, 2007; Wilhelm and Schroeders, 2019) with general intelligence as a latent variable that is constituted by dimensions of intelligences (which are also latent variables). These dimensions are then constituted by some observed variables (Carroll, 1993). The three-stratum theory of cognitive abilities is illustrated in Figure 1. Stratum II dimensions have been defined and illustrated in Table 1.

**Table 1:** Definitions of the Stratum II dimensions.

	**Label**	**Description**	**Example Task**
gf	Fluid intelligence	Reason, plan, solve abstract and complex problems; basically the ability to maintain, to mentally manipulate, and to store information; strong link with working memory capacity	Number series
gc	Crystallized intelligence	Describes the breadth and depth of cultural knowledge that is passed on to the individual through acculturation (e.g., formal learning). Is often measured with (and reduced to) verbal ability indicators, predominantly vocabulary tasks.	Vocabulary
gsm	Short-term memory (General memory and learning)	Retain and maintain a limited amount of information for a short period of time.	Memory span
gv	Visual processing (Broad visual perception)	Perceive, manipulate, store, and retrieve visual images such as shapes, forms, colors, etc., and more complex visual stimuli. This also includes spatial orientation, transformation, and moving visual objects.	Spatial relations
ga	Auditory processing (Broad auditive perception)	Analyze, manipulate, understand, and synthesize sound elements, sound groups, and sound patterns. The key feature is the cognitive control in perception of auditory material (i.e., handle the competition between signal and noise).	Speech sound discrimination
glr	Long-term memory and retrieval (Broad retrieval ability)	Store and consolidate new information in long-term memory. Fluently retrieve stored information (e.g., concepts, ideas, items, names).	Word fluency
gs	Processing speed (Broad cognitive speediness)	Perform over-learned or elementary cognitive tasks under time constraints, high efficiency (i.e., attention and focused concentration) is necessary.	Perceptual speed
gt	Reaction and decision speed	Quickly make elementary responses (i.e., simple reaction time) or several elementary responses (i.e., complex reaction time) when simple stimuli are presented.	Simple reaction task

***Source:** Adapted from Wilhelm and Schroeders (2019, p. 258)*.

Carroll's (1993) theory represents the key theory that has underpinned the development of intelligence tests and research (Mcgrew, 2009; Keith and Reynolds, 2010; Benson et al., 2018; Wilhelm and Schroeders, 2019). Notwithstanding its comprehensiveness and utility (Gregory, 2007; Benson et al., 2018), it is important to understand that the three-stratum theory of cognitive abilities is not without limitations. First, the datasets were obtained from the minority of the world (largely non-African samples) and implied in some sense that the meaning of intelligence and its measurement remain the same across cultures. For more details see the presentation of the historical foundations of the study of cognitive abilities in Chapter Two of Carroll's (1993) work.

Sternberg (2004) identified four models of culture-intelligence relationships based on the review of research and practices as follows: (1) Model I assumes that intelligence remains the same across time and place and therefore can be measured with the same instruments; (2) Model II accepts that differences exist in the conceptions of intelligence but encourages using the same instruments to measure it in different settings; (3) Model III assumes that the dimensions of intelligence are the same across different settings but encourages the use of context-specific instruments; and (4) Model IV assumes that the dimensions of intelligence are different and therefore must be measured using different instruments. Carroll's (1993) three-stratum theory aligns with Sternberg's (2004) Model I. Sternberg (2004) further stated that Model I paradigm reflects the theoretical positions of researchers such as Jensen (1982, 1998), Eysenck (1986), and lately Cofnas (2020) as well as many neuroscientists (Deary, 2005; Deary et al., 2010). However, the Model I perspective is an erroneous one (Grigorenko et al., 2001; Holding et al., 2004; Sternberg, 2004; Matsumoto and Juang, 2007; Cocodia, 2014).

In opposition to Model I thinking, Sternberg (2004) proposed and advanced a Model III thinking instead. Model III thinking holds that though the dimensions of intelligence are the same, the instruments of measurement ought not to be the same (Sternberg, 2004). As a result, Sternberg (2004, p. 336) concluded that the “processes of intelligence are universal, but their manifestations are not.” This seems to argue against a position he later advanced that “Conventional views of intelligence favor individuals who are strong in memory and analytical abilities” (Sternberg, 2005, p. 189) as well as arguments he advanced with Kaufman earlier (Sternberg and Kaufman, 1998). Interesting is even the conclusion advanced by Grigorenko et al. (2001) which included Sternberg as the last author:

When researchers use an ability test in a given population, the researchers sometimes assume that the mental “machinery” engaged by testing is basically the same in different cultures. This assumption appears to be false: People of different cultures differ not only in the abilities and processes that researchers test for, but also in those abilities and processes that researchers test with (p. 368).

If the conventional views about intelligence emphasize memory and analytical abilities, then it is difficult to assume, at the same time, that Model III thinking is correct, that the meaning of intelligence is the same across cultures. Perhaps, a better interpretation will be to consider it in terms of Bhaskar (1998, 2008) *real, actual* and *empirical* worlds. It may be safe to say that intelligence operates at the *real world* level but the manifestations of it occur at the *empirical world* level of observations. The dimensions of intelligence (processes of intelligence) appear to operate at the *actual world* level. This means that because there are cross-cultural variations in the meaning of intelligence (processes of intelligence) at the *actual world* level, its measurement at the *empirical world* level present a slice of what intelligence may be. This is particularly problematic again when one considers Sternberg's (2004) definition of (successful) intelligence:

- the ability to achieve one's goals in life, given one's sociocultural context; (2) by capitalizing on strengths and correcting or compensating for weaknesses; (3) in order to adapt to, shape, and select environments; and, (4) through a combination of analytical, creative, and practical abilities (p. 189).

This definition may reflect intelligence at the *real world* level, but its representations (dimensions or processes of intelligence) at the *actual world* level tends to reflect the meaning of intelligence peculiar to the particular socio-cultural context. It is against this background that the purpose of this paper is essential. Thus, the current thoughts, regardless of the empirical evidence gathered, about the constitution of intelligence is still narrow and far from being representative of how others think of intelligence.

Let us be further guided by the Bradya et al.'s (2018) contention (and rightly so) that we can expand the interpretive power of psychology when we adopt culture-conscious practices in research. Similarly, Rada et al. (2018) have contended that we can only hope to have a psychology of all *Homo sapiens* if we attempt to diversify the samples studied in psychology. This is consistent with Oppong's (2019) view that psychology can only hope to be a truly global science of human nature if the current narrow perspectives about human nature are expanded to include alternative views that bring all *Homo sapiens* into the fold. Thus, the future of indigenous psychology perspective is to seek to create a more inclusive, global psychology of all *Homo sapiens*. It does not in any way suggest that the Western model of intelligence is invalid (it is valid insofar as it represents the constitution of intelligence from a subgroup of *Homo sapiens* and it works for them) but it is limited in its capacity to account for the varied representations of intelligence that other subgroups of *Homo sapiens* have. Thus, if the most comprehensive model of intelligence (Carroll, 1993) was built on studies that excluded black African samples, its interpretive power can safely be argued to be severely limited as well. Benson et al. (2018) have further raised fresh concerns about Carroll's (1993) three-stratum theory of intelligence including inadequate specification, poor reproducibility outcomes when more modern methods are applied, and limited interpretive relevance of the Stratum II factors. This is, notwithstanding, the fact that Benson et al. (2018) failed to attend to culture as far as the sample used for their study also excluded African samples. Put together, the current model of intelligence that inform research and practice (Gregory, 2007; Benson et al., 2018) presents a narrow view of what intelligence is as well as its representations. As a result, there is a need for other views that may contribute to completing the puzzle of human intelligence.

## Delineating Cognitive Abilities on the Basis of African Philosophy

I will draw on the philosophical works carried out by African philosophers about knowledge and wisdom which shares conceptual similarities with the Western construct of intelligence. It is important to remind ourselves that all of the psychological constructs are *human kinds* rather than *nature kinds* (Danziger, 1999). As human kinds, psychological constructs are the creations of people within a specific linguistic group. Sapir-Whorf hypothesis is consistent with Danziger's (1999) contention; Sapir-Whorf hypothesis is the position that human perception of reality is shaped by thought processes which are in turn influenced by language (Whorf, 1956). Simply put, “we can only think of ourselves better in the categories that we have created in our own speech community.” (Oppong, 2017b, p. 34). That there is no exact linguistic category for intelligence in African languages (Serpell, 1993; Grigorenko et al., 2001; Gyekye, 2003; Cocodia, 2014) is an indication that African thoughts about cognitive abilities are not necessarily similar to non-African perspectives. It is little wonder that Gyekye (2003) could only discuss knowledge and wisdom as the conceptual equivalents of intelligence in Africa within African philosophy.

Drawing on African proverbs and customary practices in general and more specifically the Akan cosmology, Gyekye (2003) presented a philosophical exposition on knowledge (*nimdeε*) and wisdom (*nyansa*). According to Gyekye (2003, p. 137), *nyansa* comprises:

1. the ability to think out ways of making success in one's life—to analyse and solve practical problems of life—and the ability to pay reflective attention to the fundamental principles underlying human life and experience. Wisdom thus can be practical or theoretical (philosophical).

Using the Ewe word for knowledge “*nunya*”, Gyekye (2003, p. 138) defines knowledge as the “thing observed.” Ewe is the language spoken by the people of Volta Region of Ghana as well as in Togo while Akan is the most widely spoken Ghanaian Language. Thus, knowledge means, to the African, facts derived from the careful observations of nature. The implication is that Africans value experience-based or empirical knowledge more than theoretical knowledge. Gyekye (2003) argued that though Africans have always been acute observers of nature, the “observed facts were not generally given any elaborate theoretical explanations, for Africans' interest in the practical knowledge that they might use for their own benefits.” (p. 138). It seems that a model of intelligence such as Carroll's (1993) three-stratum theory that emphasizes theoretical or abstract concepts cannot reflect what Africans formulate as cognitive abilities. A closer look at the elements of Stratum I of Carroll's (1993) theory will convince any observer that Africans are more likely to consider those indicators as irrelevant measures of *nimdeε* and *nyansa*. Given that interest affects practice and performance (Krapp et al., 1992; von Maurice et al., 2014; Wong and Wong, 2019), it is little wonder that Africans conceive intelligence in a way that is completely different form the current Western orthodoxy. Perhaps too, the construct of intelligence, as conceived in Western orthodoxy, is a specific form of what cognitive ability is while *nimde*ε and nyansa also may constitute a particular form of cognitive ability. This is because psychology generates situated knowledge and practices (Ratele, 2017a,b). Jovanović (2005) contention that the internationalization of psychology has only aimed at promoting, distributing or impose psychological knowledge of a very specific kind to other parts of the world is truer even here. Thus, it is valid to think that globalization is nothing more than projecting one local culture to the world stage (Yankah, 2012; Oppong, 2019). Indeed, in internationalizing or globalizing psychology, a very specific kind of psychological knowledge and constructs (human kinds) have been projected onto the world stage to the extent that they sublimely assume the status of mainstream consensus. It is also perhaps this emphasis on practical knowledge that may have contributed to the absence of elaborate theoretical contributions from African psychologists until this special issue was imagined and implemented.

*Nyansa*, to the African, does not only promote the well-being of the one who possesses it but it must also be deployed in ways that do not harm the others. Gyekye (2003, p. 143) intimates the following to reflect the social orientation of the *nyansa* within African philosophy:

If a person cleverly manipulates or deceives people or pursues wrongful or socially unacceptable actions, he would not—should not—in …system of values be considered a wise person, however smart or knowledgeable he might be. There is, then, a moralistic dimension to the application of (practical) wisdom. Cleverness in achieving certain ends may not amount to wisdom (p. 143).

This is a clear departure from the Western models of intelligence whose validity have been supported through their relations to individual well-being (Lo, 2017; Cofnas, 2020) rather than a communal orientation (Serpell, 1993). Most of the life outcomes which have been linked to the intelligence (academic performance, health, longevity, career success, income, etc.) promote an individual well-being and fail to take into account how these cognitive abilities are deployed to promote the social or community well-being. Thus, one would expect that an African model of valued cognitive abilities would reflect (theoretical and practical) knowledge (theoretical and practical/useful), wisdom, and awareness of social responsibility in the use of knowledge and wisdom (a duty to protect others from harm). For instance, Serpell (2011) and Grigorenko et al. (2001) have documented evidence that social responsibility is a dimension of intelligence in Zambia and Kenya, respectively.

## Synthesis of Studies on the Conceptions of Cognitive Abilities in Africa

This synthesis is inspired by a similar work done by Carroll (1993) on the conceptions of intelligence, though his work was more of a systematic review of factor-analytic studies on intelligence conducted in some Western countries. To achieve this end, I employed the systematic review research methodology to identify and evaluate field studies that have investigated the constitution of cognitive abilities in African samples. Only one researcher or screener carried out the process even though the conventional practice is to have at least two researchers who independently search and screen the literature. Nonetheless, this use of a team of screeners for systematic review is not a required PRISMA protocol (Moher et al., 2009). Systematic review methodology offers the highest form of evidence in the evidence hierarchy over and above randomized controlled trials (O. Horstick, personal communication, November 14, 2019) and serves as the basis for developing standards of practice (Russell et al., 2009). Thus, the aim of this review was to compile and analyse the existing literature for evidence on the dimensions of cognitive abilities among Africans. I systematically searched through PubMed, PsycINFO, and Web of Science as well as reference lists of all identified studies. In addition, Google Scholar was searched for gray literature. Duplicates were removed after which abstracts of the remaining were screened and assessed for eligibility. I extracted relevant data and conducted a quality assessment on the remaining studies.

The following inclusion criteria were used: (1) reports of empirical studies focusing on intelligence among Africans. (2) With respect to the study types, only field studies that were ethnographic or qualitative in nature or mixed methods studies were included. The study should not impose any a priori theory or conceptions of intelligence on the study participants. Studies using experimental and cross-sectional designs or tested an existing theory of intelligences were excluded. (3) Only studies that sought to identify the indigenous conceptions or implicit theory of intelligence were included. (4) Only studies published after 1990 were included. I carried out the literature search until February 22, 2020. The search terms used include the following: Africa, Sub-Saharan Africa, intelligence, cognitive abilities, and abbreviations such as IQ. The literature search was managed using the reference manager, Zotero.

**Figure 2:**
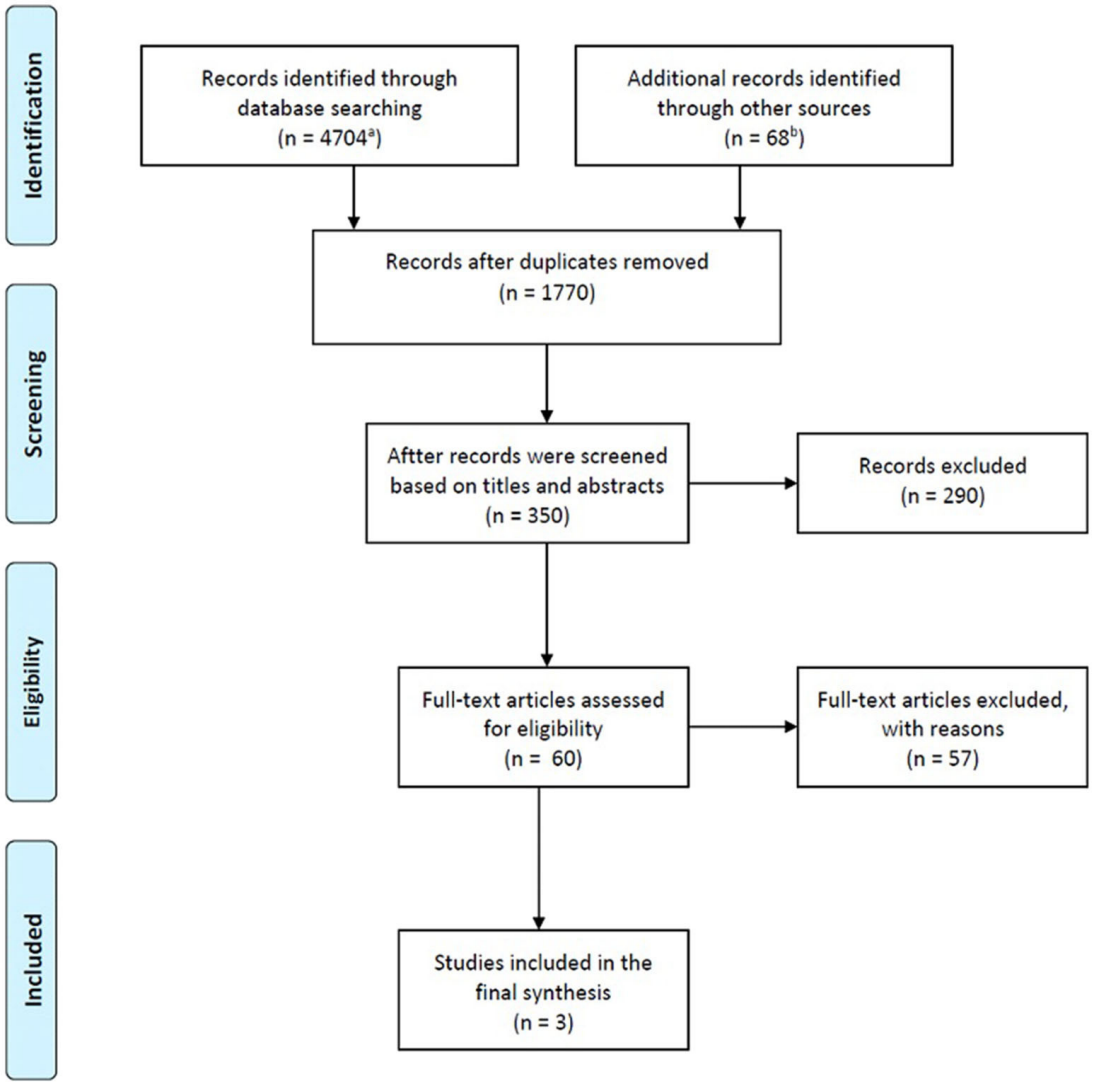
Flowchart of the literature search process. ^a^See Table 3 for details. ^b^Included those identified from the references and those identified through screening the first 100 hits of the various word combinations.

Four thousand seven hundred four (4,704) articles were identified from the different databases for assessment (Figure 2). After removing duplicates, 60 were left for further analysis. An additional 68 studies were identified from the reference lists of the above articles. In addition, Prof. Emeritus Robert Serpell was consulted for additional studies and it yielded three additional studies (R. Serpell, personal communication, 24 February 2020); the studies were: Jukes et al. (2018), Neto et al. (2009), and Noyau and Gbeto (2004). Applying full inclusion and exclusion criteria on these 60 studies, 3 met the pre-specified eligibility criteria (see Figure 2). Common reasons for the exclusion of potentially relevant studies included the following: dealing with security intelligence, cognitive abilities in animals, review articles, conducting factor analysis using conventional intelligence tests, correlational studies using conventional intelligence tests, and comparative studies which used conventional intelligence.

The following articles were excluded: Fortes's (1938) study among the Tale in Ghana, the study among the Djerma-Songhali in Niger by Bissiliat et al. (1967), the Baganda in Uganda (Wober, 1974), the Ba-Bemba in northern Zambia (Kingsley, 1977), the Kipsigis in Kenya (Super, 1983), the Baoule in Ivory coast (Dasen et al., 1985) and Rwandese (Mukamurama, 1985). Apart from lying outside of the timeframe for the literature search (1990–2020), Serpell (1989) synthesized evidence from these studies, making it unnecessary to include them in another synthesis. Again, studies by Jukes et al. (2018), Neto et al. (2009), and Humble et al. (2018) were also excluded. This is because Jukes et al. (2018) started with an a priori theory of implicit theory of intelligence based on the previous studies (e.g.,: Serpell, 1993; Grigorenko et al., 2001) whereas Neto et al. (2009) employed Gardner's theory of multiple intelligences as the theoretical framework. On the other hand, Humble et al. (2018) also sought to identify the most efficient conventional IQ test capable of identifying intellectual potential; they evaluated the Ravens Standard Progressive Matrices Plus Version (SPM), the Matrix Reasoning test from the Wechsler Abbreviated Scale of Intelligence—Second Edition (WASI-II) and the Naglieri Nonverbal Ability test (NNAT2). Thus, they also cast their study within an existing theoretical framework in their ability to identify intellectual potential among poor children in Dar es Salaam.

The three (3) studies that were included in the analysis were published in 1993, 2001, and 2004. One was conducted in East Africa, Kenya (Grigorenko et al., 2001), another in West Africa, Togo (Noyau and Gbeto, 2004) and the last study in Southern Africa, Zambia (Serpell, 1993). Two of the studies were written in English Language (Serpell, 1993; Grigorenko et al., 2001) while one was in French Language (Noyau and Gbeto, 2004). All three studies employed ethnographic techniques that did not impose the researchers' conceptions of intelligence on the participants. All of the studies attempted to identify the implicit theory of intelligence from the perspective of the participants. Two of the studies identified three elements of intelligence (Serpell, 1993; Noyau and Gbeto, 2004) while one yielded two elements (Grigorenko et al., 2001). However, even the one with two major dimensions (Grigorenko et al., 2001) first identified three elements given that *rieko* was also considered the umbrella term for the other components.

That only three studies met the criteria is not surprising because conducting a study to unveil the implicit theory of cognitive abilities often require deploying anthropological research methodology, a skill that is often lacking among psychologists. Besides, it is also very time-consuming. It can be concluded from the analysis that an African conception of cognitive abilities has about three components, namely: cognitive competence (analytical ability and expertise), wisdom, and socio-emotional competence.

In his analysis of the typology of dreams, Nwoye (2017) integrated the typologies reflective of African dreams with Euro-American conception of dream. This was done to advance the argument that Western perspectives on human nature are legitimate but insufficient to account for the experiences of all *Homo sapiens* (Nwoye, 2017; Bradya et al., 2018; Rada et al., 2018; Oppong, 2019). Owing to the fact that an indigenous approach to psychology ought to strive to contribute to global psychological science (Brzezinski, 2014; Grzelak, 2014; Oppong, 2019), I attempted to integrate relevant elements of Carroll's (1993) three-stratum theory with the findings of the synthesis of the available evidence on Africans' implicit theory of intelligence. First, the Stratum I elements are not very good instances of intelligence to the African as they do not have inherent practical values; African philosophers have revealed that human cognitive abilities always serve a practical purpose more than just theoretical or abstract desires (see Gyekye, 2003).

Grigorenko et al. (2001) illustrated this emphasis on the practical value of intelligence in the African cosmology when she and her colleagues tried to reveal the interrelatedness of the components of intelligence among the Luo speakers in Kenya. Grigorenko et al. (2001) wrote:

A boy wakes up in the morning and realizes that there is no food for the coming days at home, where his grandmother and a number of siblings live. He decides to go to the bush to cut firewood and burn charcoal [alternatively he goes fishing], he then transports the charcoal to a nearby town and sells it at a good profit, and takes all the money back to his grandmother.

The story is exemplary in that the boy shows *paro* (thoughtfulness and intellectual initiative) in realizing the problem and *winjo* (comprehension) in working out a solution; he displays *luoro* (consideration and respect) for his family members, and he demonstrates *rieko* (skill) in generating the desired outcome (p. 370).

The above clearly shows how Carroll's (1993) Stratum I elements are poor indicators of cognitive competence among Africans. Africans' conception of intelligence is more tied to real-life outcomes and therefore need to be assessed as such.

**Figure 3:**
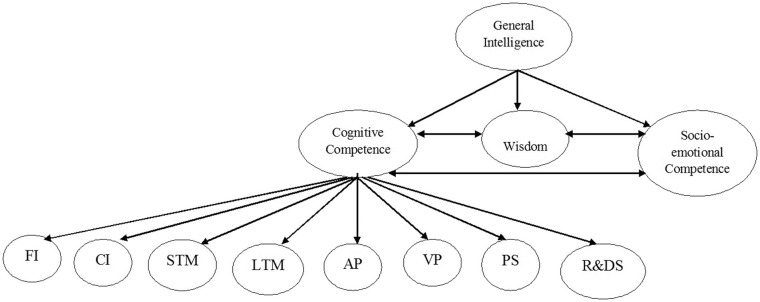
A model of valued human cognitive abilities. FI, Fluid Intelligence; CI, Crystallized Intelligence; STM, Short-term Memory; LTM, Long-term Memory; AP, Auditory Processing; VP, Visual Processing; PS, Processing Speed; R&DS, Reaction and Decision Speed.

Second, the Stratum II elements reflect common elements of the cognitive competence found in the ethnographic studies on Africans' conceptions of intelligence (see Table 2). As a result, it is retained in a theory of intelligence that reflect all of Homo sapiens but not just a sub-group. Thus, a model of valued human cognitive abilities (from an African perspective) is more likely to be as illustrated in Figure 3.

**Table 2:** Evidence table.

**1st author, year, country**	**Study design**	**Objectives**	**Population and sample size**	**Relevant Results**	**Conclusion**
Serpell (1993), Zambia	Ethnographic research and longitudinal in nature	To articulate the cultural framework within which behaviors and personalities were interpreted by the community into which the participants were born.	**Population:** A-Chewa speakers living Zambia. **Sample:** 46 children (boy = 27, girls = 19); 61 adults.	Documented three elements of the conceptions of intelligence, viz: *Nzelu* (Wisdom) *Chenjela* (Expertise) *Tumikila* (Cooperative responsibility) comprising **3a**. *Mvela* (Attentiveness, Obedience) **3b**. *Khulupilika* (Trustworthiness, Cooperation) It was documented that, as young adults, the participants ranked *ulemu* (respectfulness and compassion) above *nzelu* and its components.	Chewa speakers use *nzelu* both as the word for intelligence as well as the component of it relating to only wisdom. There appears to be three components given that *Tumikila* is an umbrella term for *mvela* and *khulupilika*.
Grigorenko et al. (2001), Kenya	Sequential exploratory mixed methods design (with ethnographic study preceding the quantitative study)	To understand and quantify the components of the Luo conception of intelligence. To understand the interrelations among these various components as they are applied to real-world individuals. To understand the relation between assessments of real world individuals via the Luo conception of intelligence and via conventional Western assessments of intelligence as well as measures of school achievement.	**Population:** Luo people living in Ugingo village in Kenya **Sample for qualitative study**: In-depth interviews with both children (n = 14) and adults (n = 13); two series of semi-structured interviews with different groups of adults (first series n = 28, second series n = 23). **Sample for quantitative study:** 86 Luo children (43 boys; 43 girls) as well as three groups of raters (peers of participants; teachers of participants; adults in the community familiar with at least three of the participants).	First, four qualities that express intelligence were identified, namely: **1**. *rieko* (translated as intelligence, smartness, knowledge, ability, skill, competence, and power) **2**. *Luoro* (social qualities, such as respect and care for others, obedience, diligence, consideration, and readiness to share). **3**. *Paro* (the thought processes required for problem-solving and the thought processes involved in caring for other people). 4. *Winjo* (abilities to process what is said or what is going on at a given point in time). Second, factor analysis also revealed two latent factors (social-emotional competence and cognitive competence). It was only *rieko* which and cognitive competence that correlated with scores on Western cognitive ability tests and English and Mathematic achievement.	Cognitive competence (*reiko* and *paro*) and social-emotional competence (*luoro* and *winjo*) appeared to drive judgements of peer, adult, and teacher ratings.
Noyau and Gbeto (2004), Togo	Mixed methods design (semi-structured interviews and administration of questionnaire)	Overall objective was to investigate organizations of intelligence in the Ewe culture (Southern Togo). Specific objectives: To identify the key terms used to conceptualize intelligence. To explore the judgements of different groups of participants about the conceptions of intelligences identified in the first part of the study.	**Population:** Ewe/Gengbe speakers in Lomé and its surroundings **Sample:** *Group 1:* 30 parents and 20 teachers interviewed in French *Group 2:* 10 illiterate parents interviewed in Ewe/Gengbe.	First, Ewe speakers tend to use more verbal predicates to express their conceptions of intelligence than nouns and adjectives given that there are fewer adjectives in the Ewe Language. Second, conceptions of intelligence are most often expressed in terms of the lexicons about parts of the body; usually, they are metaphorical expressions. They identified three types of intelligences, namely intelligence required in school, relate with others in the community, and skills to perform certain tasks. There were no nouns or adjectives to express these elements of intelligence as in the other studies given the fact that verbal predicates to express their conceptions of intelligence in the language.	Three distinct kinds of intelligence emerged: Intelligence required for schooling (pass exams, go to the next class), Intelligence required by practical life (making fire) Intelligence required for social adaptation (behaving well).

The Carroll's (1993) Stratum II elements reflect the essence of cognitive competence as found in the synthesis. As a result, the definitions provided Carroll (1993) are largely adequate even in the African context as constitutive of cognitive competence. However, wisdom and socio-emotional competence require definitions. Within this context, Gyekye's (2003) definition of wisdom is applicable here. For emphasis, I repeat his definition (Gyekye, 2003):

the ability to think out ways of making success in one's life—to analyse and solve practical problems of life—and the ability to pay reflective attention to the fundamental principles underlying human life and experience (p. 137).

Thus, wisdom is the ability to combine both cognitive competence and socio-emotional competence to successfully solve any given problem. In other words, wisdom involves demonstrating thoughtfulness, and the intellectual initiative in the realization of a problem, and having the understanding required to work out a solution while displaying consideration for one's community as well as demonstrating the skills required to generate the desired outcomes. On the other hand, socio-emotional competence relates to the display of respect and care for others, obedience towards those deserving of it, diligence, consideration, and readiness to share with one's community.

Though the model is preliminary, it suggests that general intelligence or general cognitive ability, when viewed within the African context, comprises cognitive competence, wisdom, and socio-emotional competence. These three elements of cognitive abilities are interconnected. The appropriation of Carroll's (1993) Stratum II elements into the new model is consistent with the goal of indigenous psychology Africa; indigenous psychology acknowledges that current Western situated body of knowledge in psychology is valid but limited in its capacity to account for the experiences of all humans (Oppong, 2019). Again, when African elders think of cognitive competence they mean all of the analytical abilities Carroll (1993) identified (Serpell, 1993; see Grigorenko et al., 2001). It is within this context that it becomes useful to use Carroll's (1993) representations to contribute to explaining cognitive competence in this model. Noting the model presented in Figure 3 is preliminary is important because the model was based on three ethnographic studies. More ethnographic studies on the conceptions of intelligence in Africa are needed to validate the model presented in Figure 3. A more recent ethnographic study is Dzokoto's (2020) but it investigates the constitution of the mind rather the conceptions of intelligence. Now let us turn our attention to the implications of this model in both practice and research.

## Implications for Practice and Research

If we consider the model (illustrated in Figure 3) as valid based on the ethnographic evidence presented in Table 3, we might also want to consider how this should influence practice. For instance, Carroll's (1993) theory has influenced research and practice in the domain of intelligence testing in the Euro-American practice (Gregory, 2007; Benson et al., 2018; Wilhelm and Schroeders, 2019). As a result, the implications of the model of valued cognitive abilities for both research and practice are presented. First, I discuss the implication for research because an evidence-informed practice will improve intelligence testing in Africa and the rest of the world. Though this model has been developed on the basis of the available psycho-ethnographic evidence, more studies need to be conducted. For instance, if the Stratum I elements in Carroll's (1993) theory lack inherent practical values that the African communities attach to intelligence, then there is a need for psychometricians to begin to develop indicators that are more concrete or real-life-like than the abstract indicators in the conventional or Western intelligence scales. As Opoku (2012) argued, the same students who fail tests on mathematical problems presented in abstract forms in school perform complex mathematical calculations in the real life on the streets; this is another instance that emphasizes the practical value attached to intelligence by Africans.

**Table 3:** Details of the results of the literature search from the databases using the various combinations of the search terms.

**PubMed**	**Web of Science**	**PsychINFO (APA PsycNET)**
Africa and … IN – 2225 IQ – 160 CA – 139 Sub-Saharan Africa and … CA – 65 IN – 1597 IQ – 67	Africa and … IN – 326 IQ – 29 CA – 54 Sub-Saharan Africa and … CA – 6 IN – 14 IQ – 4	Africa and … IN – 14 IQ – 2 CA – 2 Sub-Saharan Africa and … CA – 0 IN – 0 IQ – 0

*IN, Intelligence; CA, Cognitive abilities*.

With respect to crystallized intelligence, Grigorenko et al. (2001) developed, in their study, the 33-item DhoLuo Vocabulary Scale as a measure of crystallized intelligence based on the language spoken in the home and community (that is DhoLuo) and the children were then asked to provide synonyms for each word. This approach may be useful for consideration for adoption in testing practices in various settings, particularly when life-changing decisions are to be made about people. Alternatively, the vocabulary that inhere in the local variety of English or any other adopted non-native language could be useful. Dako (2012, p. 1483) argues that the local variety of a non-native language “does not refer to a non-standard variety… but to a local variety that has to some extent indigenised itself to incorporate certain distinct phonological, lexical, pragmatic and structural features.” Thus, there are varieties of English or French spoken in different parts of the world and they are all standard versions which have indigenised themselves to the localities.

Attention needs to be given to the dimensions of wisdom and socio-emotional competence. A more appropriate approach will be to develop instances of situational decision-making vignettes to which the testees will respond. These vignettes should be taken from the everyday living experiences of the testees. This is, therefore, a call on psychologists in Africa to begin to collect qualitative data about wisdom or derive from appropriate sources such as proverbs and wise-sayings to prepare items for such measures which can be validated on a national norm in the respective countries. Where appropriate, ratings by peers or adults in the community with respect to the testee's ability to exhibit wisdom and socio-emotional competence should be considered (Serpell, 1993; Grigorenko et al., 2001). An alternative will be to develop a personality test that measures these attributes. However, this may suffer from the bias of social desirability. Again, further research is needed to investigate whether data on African samples fit the model. This will help fine-tune it to increase its utility in research and practice.

Given that there will also be contextual differences always requiring adapting the scales, it would appear that cross-cultural/national testing and comparison will be difficult. Yes! It will be difficult and for the apparent reasons presented above. Perhaps, measuring and comparing cognitive abilities across countries or ethnic groups is not possible as they would never yield meaningful data that reflect the true meaning of intelligence in every group. Measuring and comparing cognitive abilities across countries or ethnic groups constitutes an instance of internationalizing psychology. However, “stubbornly” continuing the practice of measuring and comparing cognitive abilities across countries or ethnic groups is problematic; continuing to do so without the appropriate steps to use measures that reflect the true meaning of intelligence to each group further affirms Jovanović (2005, p. 78) contention—that internationalization of psychology has sought “to promote, distribute or impose psychological knowledge of a very specific Western territorial and cultural origin to the other parts of the world territory and socio-cultural landscape.” It is unhealthy when life-altering decisions are made about other people's life on the basis of such instruments.

In absence of appropriate instruments to measure crystallized intelligence (cultural knowledge), wisdom and socio-emotional competence, practicing psychologists involved in assessment should interview parents and significant others about their judgement of the testees on these attributes. Another critical question to ask is: how does a clinician identify learning disabilities in children if there cannot exist common tests of cognitive abilities? The question to ask rather is: must we define learning disabilities with reference to school work? The current thinking about learning disabilities is school-based and it is said that it occurs in individuals who otherwise exhibit at least average abilities essential for thinking and or reasoning (Us National Joint Committee on Learning Disabilities, 2011). How will a clinician evaluate a child fluent in Setwana (in Botswana) or Akan (in Ghana) with difficulty speaking English after having had the necessary learning experiences? When crystallized intelligence or cultural knowledge (often measured as knowledge of vocabulary in a given European Language) is utilized, it assumes that English Language (or Portuguese or French) is the child's native language when it is either Setwana or Akan or Ewe. These and other practices that rely on the use of tests of cognitive abilities ought to be based on a revised understanding of intelligence. Instances used to measure cognitive abilities should be more of the concrete tasks than abstract tasks in the African context. This will help us avoid misdiagnosing people and prescribing interventions, be it at the community level or the individual level (Oppong, 2015, 2019).

## Conclusion

It is perhaps important to conclude that the available ethnographic data changes how we ought to conceptualize intelligence or cognitive abilities in general and indicators used to measure them. That wisdom and socio-emotional competence do not form part of Carroll's (1993) three-stratum theory intelligence may be revealing; psychology imported from the West comes along with the hidden cultural orientation of the producers of the knowledge. The Serbian psychologist Gordana, Jovanović (2005) and the German psychologist Irmingard Staeuble (2005) maintain that psychology is exported from the US and Western Europe along with cultural values such as individualism or an emphasis on the individual. It is little wonder that the Euro-American indigenous theory of intelligence does not include socio-emotional competence and wisdom. African psychologists, worldwide, are being called upon to collaborate more with cultural anthropologists and African philosophers to further explore African implicit theories of intelligence. In this direction, a book project requiring African philosophers deploying their ethno-philosophies to examine theories of intelligence will serve psychology well. It may, perhaps, be a plausible move on the part of African psychologists to cajole their philosopher counterparts to begin to look closely at intelligence and other psychological constructs in order to provide the philosophical basis for psychological research and practice.

## Data Availability Statement

Publicly available datasets were analyzed in this study. This data can be found here: Pubmed, Web of Science, PsycINFO, Google Scholar.

## Author Contributions

The author confirms being the sole contributor of this work and has approved it for publication.

## Conflict of Interest

The author declares that the research was conducted in the absence of any commercial or financial relationships that could be construed as a potential conflict of interest.
